# Twelve Months of Time-restricted Eating and Resistance Training Improves Inflammatory Markers and Cardiometabolic Risk Factors

**DOI:** 10.1249/MSS.0000000000002738

**Published:** 2021-07-09

**Authors:** TATIANA MORO, GRANT TINSLEY, FRANCESCO Q. PACELLI, GIUSEPPE MARCOLIN, ANTONINO BIANCO, ANTONIO PAOLI

**Affiliations:** 1 Department of Biomedical Sciences, University of Padova, Padova, ITALY; 2 Department of Kinesiology and Sport Management, Texas Tech University, Lubbock, TX; 3 Department of Psychology, Educational Science and Human Movement, University of Palermo, Palermo, ITALY

**Keywords:** INTERMITTENT FASTING, RESISTANCE EXERCISE, BODY COMPOSITION, INFLAMMATION, METABOLISM, HEALTHY SUBJECTS

## Abstract

**Introduction:**

Recently, a modified intermittent fasting protocol was demonstrated to be able to maintain muscle mass and strength, decrease fat mass, and improve some inflammation and cardiovascular risk factors in healthy resistance-trained males after 2 months. The present study sought to investigate the long-term effects on these parameters.

**Methods:**

The experiment was a single-blind randomized study. Twenty healthy subjects were enrolled and underwent 12 months of either a time-restricted eating (TRE) diet or a normal diet (ND) protocol, along with resistance training. In the TRE protocol, subjects consumed their energy needs in three meals during an 8-h period of time each day (1 pm, 4 pm, and 8 pm). Subjects in the ND group also had three meals, which were consumed at 8 am, 1 pm, and 8 pm. Groups were matched for kilocalories consumed and macronutrient distribution at baseline.

**Results:**

After 12 months of TRE, body mass, fat mass, insulin-like growth factor 1, and testosterone were significantly lower compared with ND. Moreover, inflammatory markers (interleukin 6, interleukin 1β, and tumor necrosis factor α), insulin sensitivity (fasting glucose, insulin, and homeostatic model assessment for insulin resistance index), and lipid profile (cholesterol, HDL, and LDL) significantly improved after TRE compared with ND. Finally, subjects in TRE spontaneously decreased their daily energy intake, whereas those in ND maintained their starting kilocalories per day. No adverse events were reported.

**Conclusions:**

Our results suggest that long-term TRE combined with a resistance training program is feasible, safe, and effective in reducing inflammatory markers and risk factors related to cardiovascular and metabolic diseases.

## Abstract 2

Supplemental digital content is available in the text.

Over the past century, lifestyles and nutritional habits have rapidly changed, contributing to increased obesity, metabolic syndrome, and risk of cardiovascular diseases and diabetes (1,2). As well documented by several epidemiological studies, lifestyle has a huge effect on the development of chronic disease, such as cardiovascular disease, metabolic syndrome, and diabetes (2,3). The successful implementation of physical activity with a proper nutritional plan has been recognized as one of the main strategies to counteract the evolution of chronic disease and mortality (4–6).

Recently, caloric restriction (CR) and intermittent fasting (IF) have been promoted as novel dietetic approaches to reduce obesity and improve many health-related outcomes. CR seems to be more efficient for reducing body fat, whereas both CR and IF improve blood pressure, lipid profile, and insulin resistance in overweight subjects (7–9). The positive effects of IF and CR seem to be primarily driven by a reduction in the oxyradical production and an increase in cellular stress resistance, which are in most pathways similar to the mechanism observed during regular physical exercise (10). Moreover, 16 h of fasting are able to promote cellular autophagy, which is a crucial intracellular process to preserve cell function, mediating protein degradation, and cytoplasmic recycling (11–13).

However, in a review of the literature, Barnosky et al. (14) observed that neither CR nor IF had clinically significant effects on glucose levels if the diet was followed for 3 to 24 wk. Indeed, a majority of the studies performed on CR or IF were short term (≤6 months), and one of the major problems of this dietetic regimen is the long-term adherence (15).

Time-restricted eating (TRE) is a less extreme form of IF, in which the whole energy intake occurs in a definite window of time (normally 8–12 h) during the day. We have recently shown that 8 wk of TRE combined with resistance training reduced fat mass (FM) and proinflammatory markers (i.e., interleukin (IL) 6, tumor necrosis factor α (TNF-α)), and enhanced some health-related biomarkers (i.e., insulin, HDL, triglyceride (TG)) without affecting muscular performance (16–18). Confining the access to food to 8–12 h·d^−1^ seems to influence circadian rhythms and energy metabolism through the combined effects of leptin, adiponectin, and insulin (11,19). The synergistic interaction between the feeding/fasting state also regulates the anabolic and catabolic signal throughout the day and coordinates the circadian rhythm of different organs, thereby supporting the prevention, and sometimes the reversal, of metabolic diseases (20–22). Recently, using an animal model, Yasumoto et al. (23) showed that altering the feeding/fasting cycle can desynchronize peripheral clocks and promote obesity and metabolic disorders. Thus, it could be possible that altering the feeding window may affect the circadian clocks, influencing not only the expression of specific genes (such as BMAL1 and CLOCK) on the hypothalamus (24,25) but also the production of several hormones involved in the appetite control. Because prevention of weight gain in nonobese subjects is an important strategy to reduce the incidence of many chronic diseases such as diabetes, cardiovascular disease, cancer, and dementia (26), we aimed to examine the effects of a long-term (12-month) TRE pattern on body composition, metabolic, and cardiovascular risk markers. We hypothesized that 12 months of TRE protocol would lead to lower body mass and a reduction in inflammatory and metabolic markers as compared with a normal dietary pattern.

## SUBJECTS AND METHODS

### Overview

We performed a follow-up analysis on a study recently published from our group (16), which compared the effect of a particular IF protocol, in which all calories are consumed in an 8-h window (TRE), with a normal meal distribution regimen (ND). The intent of the current study was to explore the long-term effect of TRE on health-related biomarkers. The experiment was single-blind randomized study; all subjects received an unique code to maintain blinding of researchers. The study was retrospectively registered at ClinicalTrials.gov as NCT04668378. This study was approved by the ethical committee of the Department of Biomedical Sciences (institutional review board no. HEC-DSB 5/13), University of Padova, and conformed to standards for the use of human subjects in research as outlined in the current Declaration of Helsinki. The following primary outcome measures were FM and fat-free mass (FFM). Secondary outcome included lipid profile, insulin sensitivity markers, and muscle strength measured via one repetition maximum (1RM) test.

### Participants

After completion of the first 8 wk of intervention, subjects were asked to volunteer to continue the intervention for 10 extra months. A regular practice of resistance training for at least 5 continuously years was the main inclusion criterion of the study; whilst; the usage of steroids or any clinical problems were considered an exclusion criterion. Twenty-six of the 34 participants agreed to continue the assigned nutritional and training protocols as previously described (16) using a random sequence generator (https://www.graphpad.com/quickcalcs/randomize1/) and based on a 1:1 allocation ratio. Six subjects withdrew during the first month; thus, 10 subjects for each group completed the study throughout the year. Moreover, one subject from the ND group did not attend the body composition and strength tests and was therefore not considered for this analysis (Figure, Supplemental Digital Content 1, CONSORT diagram, http://links.lww.com/MSS/C363). Because the sample is a subgroup of the previous study, data for the subjects who completed the 12-month period are presented at baseline (pre), at 2 months (2 months) extrapolated from Moro et al. (16), and at the end of 1 yr of intervention (12 months). All participants gave their informed written consent before their inclusion in the study. Data collection occurred from January 2014 to March 2015 at the University of Padova, Italy.

### Measurements

Complete details of study procedures can be found in Moro et al. (16). Briefly, for each time point, participants underwent three different visits, and they were asked to refrain from caffeine, alcohol, and vigorous physical activity during the previous 24 h. During the first visit, subjects arrived after an overnight fasting and blood samples were collected from the antecubital vein. Immediately after, subjects lied supine in a quiet and approximately 24°C room for 15 min before measuring respiratory gas parameters. Before each test, the calorimeter was calibrated as per the manufacturers’ instruction. Oxygen uptake (V˙O_2_) and carbon dioxide production (V˙CO_2_) were collected for 30 min via a standard open-circuit calorimetry (max Encore 29 System, Vmax; Viasys Healthcare, Inc., Yorba Linda, CA) with breath-by-breath modality. The last 20 min of the trial was used to calculated resting energy expenditure (REE) and respiratory ratio (RR) using the modified Weir equation (27).

The following day, subjects underwent body composition analysis assessed by dual-energy x-ray absorptiometry (QDR 4500 W; Hologic Inc., Arlington, MA), which was used to obtain FM, FFM, and an estimation of the visceral adipose tissue (VAT). Moreover, limb muscle area was calculated using a previously (28) validated software (Fitnext®; Caldogno, Vicenza, Italy) combining thigh and arm skinfolds with the correspondent circumferences. The same operator (A.P.) obtained midarm and midthigh circumferences using an anthropometric tape and measured biceps, triceps, and thigh skinfolds with a Holtain caliper (Holtain Ltd, UK).

Muscle strength was measured on separate days using the 1RM test for leg press and bench press exercises. For each exercise, subjects performed five repetitions with their normal 10RM as a warm-up routine; the load was then gradually increased until participants reached exhaustion. The weight loaded for only one repetition was considered as 1RM. The 1RM was also assessed throughout the 12 months to ensure the necessary weight adjustment to maintain a proper intensity during training sessions (data are not presented).

### Training

All participants had at least 5 yr of uninterrupted resistance training experience before study commencement. After the first 2 months of supervised training, subjects maintained a thrice-weekly training schedule. For the remaining 10 months of study, training sessions were not directly supervised because of the high experience of subjects. Training intensity fluctuated during the experimental period between 75% and 90% of 1RM to alternate strength and hypertrophy training cycles. Training sessions were performed between 4:00 and 6:00 pm to fall within the eating window for both groups

### Diet

All subjects completed a validated 7-d food diary (29) before starting the intervention (pre), and during the last week of the second (2 months) and twelfth months (12 months). Diet composition and macronutrient distribution during all experimental time points for both groups are presented in Table 1. As per Moro et al. (16), participants were instructed to not alter their usual caloric intake or diet composition. Each subjects received a personalized diet protocol based on the analysis of the food diary at baseline (pre) to facilitate meals organization and caloric distribution throughout the day. The TRE group was asked to consume the total caloric needs divided into three meals eaten in an 8-h window (~1 pm, 4 pm, and 8 pm) and fasted for the remaining 16 h. The ND group maintained a normal meal distribution, as the total energy intake was divided into three meals consumed in approximately 12-h window (~8 am, 1 pm, and 8 pm).

**TABLE 1:** Diet composition and macronutrients distribution during the experimental period in both groups.

	TRE (*n* = 10)			ND (*n* = 10)			2-Way ANOVA Time–Diet
	Pre	2 Months	12 Months	Pre	2 Months	12 Months	
Total (kcal·d^−1^)	2760.7 ± 282.1	2750.0 ± 303.9	2580.0 ± 245.3*^,^**	2942 ± 279.6	2996.0 ± 267.8	2978.0 ± 245.1***	0.0287
Carbohydrates (kcal·d^−1^)	1466.5 ± 162.3	1381.2 ± 183.5	1374.5 ± 132.5*	1548.5 ± 179.9	1616.2 ± 164.0***	1606.2 ± 152.6***	0.0027
Fat (kcal·d^−1^)	698.3 ± 121.1	752.8 ± 180.8	588.0 ± 79.8**	787.0 ± 152.0	734.4 ± 83.6	695.9 ± 91.8	n.s.
Protein (kcal·d^−1^)	595.9 ± 75.6	616.2 ± 67.8	617.6 ± 62.9	606.6 ± 78.3	645.8 ± 49.7	675.9 ± 72.8*	n.s.
Protein (g·kg^−1^ BW)	1.79 ± 0.19	1.86 ± 0.18	1.92 ± 0.13	1.79 ± 0.18	1.90 ± 0.09	1.93 ± 0.13	n.s.
Carbohydrates (%)	53.1 ± 1.4	50.2 ± 3.9	53.3 ± 1.6**	52.6 ± 2.7	53.9 ± 1.6***	53.9 ± 1.6	0.0250
Fat (%)	25.3 ± 3.1	27.3 ± 4.0	22.7 ± 2.1**	26.6 ± 4.0	24.4 ± 1.3	23.4 ± 2.4	n.s.
Protein (%)	21.6 ± 2.7	22.5 ± 3.0	24.0 ± 1.3*	20.8 ± 2.7	21.7 ± 1.5	22.7 ± 1.9	n.s.

Results are presented as mean ± SD.

*Statistically different from the Pre value (*P* < 0.05).

**Statistically different from the value at 2 months (*P* < 0.05)

***Statistically different from TRE value (*P* < 0.05).

n.s., not statistically significant.

In the TRE group, calorie distribution was 40% at breakfast (which was the first meal that actually “break the fast”), 25% at lunch (or second meal), and 35% at dinner, whereas ND subjects consumed, respectively, 25%, 40%, and 35% throughout the three meals. Subjects consumed 20 g of whey proteins 30 min after each training session. During the first 2 months, subjects were contacted weekly by a dietitian to ensure the adherence to the diet protocol, whereas throughout the remaining year of intervention, subjects were contacted less closely, approximately every month. During the interviews, the dietitian asked information about meal routines (primarily meal timing and composition), appetite, and any difficulties encountered in maintaining the diet protocol. When necessary, advice was given on how to prepare meals or change the composition of ingredients to try to keep adherence to the protocol as close possible. Subjects were considered not compliant to the TRE protocol if they did not follow the given meal timing for 3 consecutive days or for more than 35 d throughout the 12 months of intervention (10% of the total time).

### Laboratory analyses

Blood samples taken from the antecubital vein and aliquoted and stored at −80°C upon centrifugation (4000 RPM at 4°C using centrifuge J6-MC by Beckman). Samples from all time points were measured in duplicate, and one sample was chosen as internal standard to assess variation when the reagent lot between analyses was different.

Insulin-like growth factor 1 (IGF-1) was measured using a sandwich immunoassay and analyzed with Liaison XL (DiaSorin S.p.A, Vercelli, Italy). The reference range of the test differed based on age (coefficient of variation (CV), 5.6%–9.6%). An electrochemiluminescent detection method was used to test plasma testosterone using Testosterone II (Roche Diagnostics, Indianapolis, IN) on Modular Analytics E 170 analyzer. An automated chemiluminescence method (ACS 180 SE; Bayer, Milan, Italy) was used to detect thyroid-stimulating hormone (TSH) and free triiodothyronine (T3). The interassay CV values were 10% for testosterone and 8.6% for TSH.

Leptin and adiponectin were measured by radioimmunoassay using commercially available kits (Leptin: Mediadiagnost; Adiponectin: DRG Diagnostic; CV, 5%–10%); the ratio between leptin and adiponectin concentration was used to determine the leptin/adiponectin ratio (LAR) index.

Glucose was assessed using the glucose oxidase method (glucose analyzer; Beckman Instruments, Palo Alto, CA; CV, 1.2%), whereas a chemiluminescent immunoassay (Siemens Immulite 2000; CV <6%) was used to detect insulin concentration. The homeostatic model assessment for insulin resistance index (HOMA-IR) was calculated using the formula: insulin (mU·L^−1^) × glucose (mg·dL^−1^)/405.

An enzymatic colorimetric method using a Modular D2400 (Roche Diagnostics, Basel, Switzerland) was applied to detect total cholesterol, HDL cholesterol, LDL cholesterol, and TG concentration. LDL fraction was calculated using the Friedewald’s formula: LDL = Total Cholesterol − HDL − (TG/5).

The interassay CV values were 2.9% for total cholesterol, 1.85% for HDL, and 2.4% for triacylglycerol concentrations.

IL-6, TNF-α, and IL-1β were measured using Quantikine HS Immunoassay Kit (R&D Systems, Minneapolis, MN). The interassay CV ranges were 3.5%–6.2% for IL-6, 3.2%–6.3% for TNF-α, and 3.2%–6.3% for IL-1β.

### Statistical analysis

An *a priori* power analysis was performed for the 2 months of intervention (G*Power, v.3.1.9.2) as previously described (16). FM was specified as the primary dependent variable; thus, using an *α* risk of 0.05 and power of 0.8, it was estimated that 15 participants were needed to detect significant changes in FM. Subjects have voluntarily continued the intervention for 10 extra months; therefore, we performed a posteriori power analysis. With 10 subjects per group and an *α* risk of 0.05, we achieved a power of 0.797 using FM as the primary outcome. Results are presented as mean ± SD. Continuous variables were tested for normality using the Shapiro–Wilk test. A two-way repeated-measures analysis of variance (ANOVA) with Bonferroni’s posttest was used to assess time-dependent changes and differences between the groups at each time point. For each group, Cohen’s *d* effect size was assessed by dividing the difference between baseline and 12 months by the pooled SD. Association between variables was analyzed with a two-tailed Pearson correlation. *P* values of <0.05 were considered statistically significant. Statistical analyses were performed using GraphPad Prism 8.1.2 for Mac OS X (GraphPad Software).

## RESULTS

### Long-term TRE reduced energy intake

Compared with ND, participants in the TRE group significantly reduced total energy intake by 6.4% (*P* < 0.0001) from baseline driven by a reduction of 6.1% (*P* = 0.049) in the energy derived from carbohydrates. The kilocalories derived from fat was significantly reduced between 2 and 12 months of TRE (*P* = 0.002), whereas no significant difference was observed in the ND group. Protein intake was maintained throughout the intervention and between groups (Table 1).

### TRE and body composition

Body composition results are presented in Table 2. A significant time–treatment interaction (*P* < 0.001) was observed in body mass, FFM, and FM. After 12 months, the TRE group significantly decreased total body mass (−3.36%, *P* = 0.001, 12 months vs pre; *d* = −0.27) and FM (−11.81%, *P* = 0.03, 12 months vs pre; *d* = −0.34). On the other hand, the ND group gained FFM (+2.86, *P* = 0.03, 12 months vs pre; *d* = 0.33) which induced an increase in body mass (+3.37%, *P* = 0.002, 12 months vs pre; *d* = 0.27). The TRE group decreased the VAT measure via dual-energy x-ray absorptiometry (VAT) by approximately 18.8%, whereas no change was observed in the ND group; however, these differences were not statically significant (two-way ANOVA, *P* = 0.43; time, *P* = 0.07; diet, *P* = 0.39).

**TABLE 2:** Body composition, strength and resting metabolism results.

	TRE (*n* = 10)			ND (*n* = 10)			2-Way ANOVA Time–Diet [*η*^2^]	Time [*η*^2^]	Diet [*η*^2^]
	Pre	2 Months	12 Months	Pre	2 Months	12 Months			
Body weight (kg)	83.22 ± 5.92	82.89 ± 6.04	80.33 ± 4.76*^,^**	84.64 ± 5.76	85.00 ± 5.40	87.38 ± 4.39*^,^**^,^***	<0.0001 [0.05]	0.980 [<0.01]	0.152 [0.10]
FFM (kg)	73.43 ± 4.08	73.90 ± 4.64	72.22 ± 4.40	74.17 ± 2.64	74.72 ± 2.61	76.20 ± 3.53*	0.002 [0.30]	0.394 [0.36]	0.0.449 [0.06]
FM (kg)	9.79 ± 3.32	8.99 ± 2.39	8.11 ± 1.61*	10.47 ± 3.95	10.28 ± 3.75	10.55 ± 3.75	0.015 [0.22]	0.394 [0.05]	0.449 [0.03]
VAT (cm^3^)	198.20 ± 75.03	173.90 ± 51.50	147.70 ± 28.57	200.30 ± 64.71	204.33 ± 61.10	192.68 ± 21.57	0.518 [0.04]	0.060 [0.16]	0.195 [0.10]
Arm muscle CSA (cm^2^)	48.50 ± 2.00	48.93 ± 2.31	46.40 ± 2.47*^,^**	47.70 ± 2.51	49.00 ± 2.33	52.89 ± 2.00*^,^**^,^***	<0.0001 [0.76]	0.001 [0.35]	0.044 [0.17]
Thigh muscle CSA (cm^2^)	146.21 ± 5.67	147.13 ± 6.09	141.91 ± 6.09*^,^**	145.66 ± 7.97	149.03 ± 9.12	153.70 ± 9.02*^,^**^,^***	<0.0001 [0.19]	0.025 [0.61]	0.275 [0.07]
Bench press 1RM (kg)	102.40 ± 8.04	106.80 ± 7.84	118.00 ± 7.30*^,^**	108.44 ± 9.32	111.11 ± 11.14	118.25 ± 7.74*^,^**	0.791 [0.01]	<0.0001 [0.82]	0.569 [0.02]
Leg press 1RM (kg)	271.00 ± 12.87	280.50 ± 12.12*	312.00 ± 12.06*^,^**	278.89 ± 15.37	291.11 ± 13.87*	320.63 ± 9.43*^,^**	0.740 [0.02]	<0.0001 [0.65]	0.087 [0.16]
REE (kcal·d^−1^)	1879.1 ± 118.2	1895.9 ± 142.1	1792.8 ± 76.5*^,^**	1933.9 ± 170.9	1907.6 ± 179.9	1925.8 ± 189.6	<0.0001 [0.50]	0.004 [0.27]	0.422 [0.04]
REE/BW (kcal·d^−1^·kg^−1^)	22.60 ± 0.82	22.89 ± 0.92	22.36 ± 1.11**	22.83 ± 0.87	22.41 ± 0.94	22.18 ± 1.23*	0.029 [0.16]	0.003 [0.28]	0.739 [0.01]
RR	0.84 ± 0.01	0.83 ± 0.01*	0.79 ± 0.02*^,^**	0.83 ± 0.02	0.83 ± 0.02	0.83 ± 0.01***	<0.0001 [0.66]	<0.0001 [0.65]	0.443 [0.03]

Results are presented as mean ± SD.

*Statistically different from the Pre value (*P* < 0.05).

**Statistically different from the value at 2 months (*P* < 0.05).

***Statistically different from the TRE value (*P* < 0.05).

A significant time–treatment interaction (*P* < 0.001) was observed in muscle cross-sectional area (CSA). No significant effect was found between baseline and 2 months of intervention; however, at the 12-month assessment, arm CSA in the TRE group decreased by 4.31% (*P* = 0.003, *d* = −0.47) and thigh CSA decreased by 2.90% (*P* = 0.03, *d* = −0.37), whereas CSA increases of 11.87% (*P* < 0.0001, *d* = 1.15) and 6.94% (*P* < 0.0001, *d* = 0.47), respectively, were observed with ND.

These changes in muscle mass did not affect muscle strength measures on 1RM test for the upper and lower body. After 12 months, both groups significantly increased their bench press performance (TRE: +15.42%, *P* < 0.0001, *d* = 1.02; ND: +10.82%, *P* < 0.0001, *d* = 1.64) and their leg press strength (TRE: +15.24%, *P* < 0.0001, *d* = 0.77; ND: +14.21%, *P* < 0.0001, *d* = 1.84).

### TRE reduced resting energy expenditure and the respiratory ratio

A significant time–treatment interaction (*P* < 0.001) was observed in the REE and RR adaptation (Table 2). After 12 months of TRE, we observed a significant reduction in REE (−1.5%, *P* < 0.0001, *d* = −0.44) probably due to the significant weight loss in this group; indeed, when REE was normalized for body mass, the differences from baseline were not significant (−1.1%, *P* = 0.69). No significant difference was found in the ND group for REE, whereas a significant (*P* = 0.009, *d* = −0.31) reduction at 12 months from baseline was observed for REE/BW.

TRE promoted a transition toward lipid metabolism as the RR continually decreased during the diet intervention (−5.9% 12 months vs pre, *P* < 0.001, *d* = −1.70), whereas subjects in ND maintained their RR. Together, this resulted in a significantly lower RR (*P* < 0.0001) in TRE as compared with ND after 12 months of intervention (Table 2).

### TRE reduced testosterone and IGF-1 concentrations

A significant time–treatment interaction (*P* < 0.0001) was observed for both the anabolic hormones analyzed.

Testosterone concentration was significantly reduced after 2 and 12 months of TRE diet by 3.12% (*P* = 0.01) and 16.81% (*P* = 0.0004, *d* = −0.71), respectively, whereas no change was observed in the ND group.

IGF-1 concentration was significantly reduced after 2 months of TRE intervention by 14.65% (*P* = 0.0125) and remained lower after 12 months (−14.28% from baseline, *P* = 0.020, *d* = −0.42), whereas in the ND group, the hormone level was unchanged.

Any significant changes were observed for the thyroid hormones TSH an T3 (Table 3).

**TABLE 3:** Blood parameters.

	TRE (*n* = 10)			ND (*n* = 10)			2-Way ANOVA Time–Diet [*η*^2^]	Time [*η*^2^]	Diet [*η*^2^]
	Pre	2 Months	12 Months	Pre	2 Months	12 Months			
Anabolic hormones									
Testosterone total (nmol·L^−1^)	19.55 ± 6.35	15.83 ± 3.89*	14.61 ± 0.63*	18.21 ± 5.67	18.79 ± 4.94	18.97 ± 5.35	0.002 [0.29]	0.028 [0.18]	0.327 [0.05]
IGF-1 (ng·mL^−1^)	219.70 ± 54.11	181.50 ± 30.19*	183.60 ± 31.90*	225.30 ± 36.77	227.60 ± 26.44***	223.20 ± 26.79***	0.039 [0.52]	0.042 [0.16]	0.021 [0.01]
TSH (mU·mL^−1^)	1.30 ± 0.24	1.17 ± 0.44	1.25 ± 0.26	1.29 ± 0.17	1.29 ± 0.17	1.27 ± 0.19	0.479 [0.04]	0.469 [0.04]	0.661 [0.01]
T3 (ng·dL^−1^)	83.10 ± 4.82	76.80 ± 10.26	81.60 ± 7.68	81.60 ± 4.35	82.00 ± 4.08	81.50 ± 4.67	0.153 [0.08]	0.243 [0.10]	0.553 [0.02]
Adipokines									
Adiponectin (μg·mL^−1^)	11.84 ± 1.22	13.87 ± 1.97*	14.33 ± 1.99*	10.50 ± 1.08	10.79 ± 0.88***	10.80 ± 0.68***	0.001 [0.35]	<0.0001 [0.47]	<0.001 [0.57]
Adiponectin (μg·mL^−1^·kg^−1^ FM)	1.38 ± 0.63	1.65 ± 0.54*	1.82 ± 0.38*	1.13 ± 0.41	1.18 ± 0.44	1.15 ± 0.44***	0.001 [0.22]	0.003 [0.11]	0.032 [0.51]
Leptin (ng·mL^−1^)	2.27 ± 0.65	1.81 ± 0.53*	1.63 ± 0.34*	2.30 ± 0.47	2.36 ± 0.38***	2.54 ± 0.50***	<0.001 [0.38]	0.065 [0.14]	0.017 [0.28]
Leptin (ng·mL^−1^·kg^−1^ FM)	0.24 ± 0.07	0.21 ± 0.05	0.21 ± 0.06	0.25 ± 0.12	0.26 ± 0.05	0.26 ± 0.09	0.153 [0.23]	0.163 [0.15]	0.387 [0.51]
LAR	0.20 ± 0.07	0.13 ± 0.05*	0.12 ± 0.03*	0.22 ± 0.04	0.24 ± 0.05	0.24 ± 0.05	<0.0001 [0.43]	0.001 [0.30]	0.001 [0.46]
Insulin sensitivity									
Glucose (mg·dL^−1^)	95.10 ± 5.30	86.20 ± 5.47*	86.50 ± 3.72*	95.50 ± 4.33	95.60 ± 4.65***	96.20 ± 5.01***	<0.0001 [0.57]	<0.0001 [0.53]	0.004 [0.38]
Insulin (mU·mL^−1^)	2.59 ± 0.41	1.87 ± 0.55*	1.77 ± 0.37*	2.48 ± 0.31	2.39 ± 0.45***	2.46 ± 0.38***	<0.0001 [0.45]	<0.0001 [0.51]	0.038 [0.22]
HOMA-IR	0.61 ± 0.10	0.40 ± 0.13*	0.38 ± 0.08*	0.58 ± 0.08	0.57 ± 0.12***	0.58 ± 0.09***	<0.0001 [0.56]	<0.0001 [0.60]	0.097 [0.32]
Lipid profile									
Total cholesterol (mg·dL^−1^)	194.90 ± 8.44	192.00 ± 11.08	187.80 ± 6.36	198.30 ± 13.28	197.00 ± 11.29	197.00 ± 6.62	0.289 [0.07]	0.094 [0.12]	0.144 [0.12]
HDL cholesterol (mg·dL^−1^)	53.70 ± 2.67	57.40 ± 2.46*	61.80 ± 3.97*^,^**	53.60 ± 2.07	55.10 ± 2.23	54.20 ± 2.70***	<0.001 [0.36]	<0.0001 [0.42]	<0.001 [0.54]
LDL cholesterol (mg·dL^−1^)	118.30 ± 15.08	111.80 ± 11.53	107.00 ± 7.01*	119.90 ± 11.01	117.50 ± 11.26	118.80 ± 5.33***	0.129 [0.11]	0.046 [0.16]	0.115 [0.13]
TG (mg·dL^−1^)	123.20 ± 6.94	114.00 ± 9.88*	97.10 ± 3.03*^,^**	122.10 ± 11.06	118.00 ± 11.37	120.80 ± 8.02***	<0.0001 [0.56]	<0.0001 [0.58]	0.014 [0.29]
Inflammatory markers									
IL-6 (ng·L^−1^)	1.40 ± 0.23	1.04 ± 0.20*	1.01 ± 0.28*	1.41 ± 0.40	1.33 ± 0.36	1.30 ± 0.28	0.038 [0.22]	<0.001 [0.21]	0.970 [0.00]
IL-1β (ng·L^−1^)	0.90 ± 0.18	0.78 ± 0.07	0.67 ± 0.11*	0.90 ± 0.14	0.92 ± 0.11	0.96 ± 0.14***	<0.001 [0.50]	0.031 [0.27]	0.004 [0.08]
TNF-α (ng·L^−1^)	5.40 ± 0.81	4.93 ± 0.67	4.61 ± 0.63*	5.53 ± 0.92	5.56 ± 0.78	5.34 ± 0.77	0.042 [0.27]	0.002 [0.14]	0.876 [0.00]

Results are presented as mean ± SD.

*Statistically different from the Pre value (*P* < 0.05).

**Statistically different from the value at 2 months (*P* < 0.05).

***Statistically different from the TRE value (*P* < 0.05).

### TRE reduced the LAR

The ratio between leptin and adiponectin (LAR) was significantly reduced after 2 (−29.9%, *P* = 0.0002) and 12 months (−37.0%, *P* < 0.0001, *d* = −0.82) only in the TRE group; no alterations were observed in the ND group. These results are related to a significant increase in adiponectin observed in the TRE group, respectively, of 17.4% (*P* < 0.0001) and 21.3% (*P* < 0.0001, *d* = 0.78) both at 2 and 12 months of treatment, and to a concomitant decrease in leptin concentration of 19.0% (*P* = 0.003) at 2 months and 24.9% (*P* = 0.0002, *d* = −0.64) at the end of the study.

Because these two adipokines are released from the adipose tissue, we normalized their concentration to FM, obtaining, still, the same statistical results (Table 3).

Not surprisingly, leptin concentration positively correlated with FM (*r* = 0.325; *P* = 0.01), and thus, leptin reduction correlated with body mass (*r* = −0.72, *P* = 0.0004) and FM (*r* = −0.52, *P* = 0.03) changes in response to the diets (data not shown).

### TRE and insulin sensitivity

After 2 months of TRE, glucose and insulin concentrations were significantly lower compared with baseline (glucose −9.26%, *P* < 0.0001; insulin −28.26%, *P* < 0.0001), and remarkably, these values were maintained also after 12 months of intervention (glucose −9.92%, *P* < 0.0001, *d* = −0.95; insulin −31.66%, *P* < 0.0001, *d* = −1.06), whereas no change was observed in the ND group. As a result, the HOMA-IR was reduced by 34.80% (*P* < 0.0001) and 37.83% (*P* < 0.0001, *d* = −1.32) after 2 and 12 months of TRE diet.

Interestingly, we observed a negative correlation between the basal level of adiponectin and the HOMA-IR index (*r* = −0.306, *P* = 0.02); additionally, we found that the responses of these two variables, calculated as fold change from basal, were positively associated (*r* = −0.328, *P* = 0.01), indicating that the increased level of adiponectin, due to TRE protocol, corresponded to a lower insulin resistance.

Moreover, HOMA-IR correlated with LAR value after both 2 (*r* = 0.536, *P* = 0.02) and 12 months (*r* = 0.681, *P* = 0.001) of treatment (Figure, Supplemental Digital Content 2, Pearson correlation between HOMA-IR and LAR fold change, http://links.lww.com/MSS/C364), supporting the relationship between HOMA-IR and LAR changes. Because these two variables were unchanged in the ND but decreased with the TRE protocol, it could be speculated that TRE had an important effect on insulin sensitivity management via leptin and adiponectin control. No correlation was found between HOMA-IR and VAT (*r* = 0.161, *P* = 0.26).

### TRE and lipid profile

We did not observe any significant differences in total cholesterol concentration; however, with TRE, HDL was significantly improved after 2 and 12 months of intervention (6.39%, *P* = 0.03 at 2 month; and 15.39%, *P* < 0.0001, *d* = 1.22 at 12 month) and LDL was reduced by 8.33% (*P* = 0.02, *d* = −0.51) at the end of the study. Because ND did not change the lipid profile, both HDL and LDL differed significantly (*P* < 0.0001) between groups at the 12-month assessment. Also, TG was reduced by 7.44% (*P* = 0.01) and 20.98% (*P* < 0.0001, *d* = −2.62) in the TRE group at the two assessments, with no differences observed after the ND diet.

We also observed that the reduction in TG was positively correlated with LAR changes after 12 months of intervention (*r* = 0.676, *P* = 0.001).

### TRE and inflammatory markers

A significant time–treatment interaction (*P* < 0.05) was observed for all the inflammatory markers analyzed. IL-6 decreased in the TRE group after 2 months of diet (−24.38%, *P* = 0.0008) and was kept lower throughout the end of the study (−25.22%, *P* = 0.0003, *d* = −0.75 at 12 months). IL-1β and TNF-α were significantly lower compared with baseline only after 12 months of diet; their respective concentrations decreased by 22.97% (*P* < 0.0001, *d* = −0.80) and 13.88% (*P* = 0.0008, *d* = −0.55). No significant alterations were found on the ND group.

## DISCUSSION

In this study, we investigated the effect of long-term (12 months) TRE on weight control and cardiovascular and metabolic risk factors during a resistance training protocol. After 12 months of TRE, body mass was reduced by 3.4% and inflammatory markers (IL-6, IL-1β, and TNF-α), lipid profile (HDL, LDL, TG), and insulin resistance (fasting glucose, insulin, HOMA-IR) significantly improved compared with ND without affecting muscle performance.

Is worth noting that during the 12 months of intervention, subjects in the TRE group spontaneously reduced their daily energy intake by approximately 10% without affecting protein intake, which remained around 1.9 g·kg^−1^ BW. Instead, the reductions in energy intake came from carbohydrates and fats. The observed alteration in total calories and macronutrients intake may have led to the observed diminution of the REE of approximately 5% after the TRE diet. This phenomenon has been observed also in other studies involving an alternate day IF approach, during which subjects reduced their caloric intake by approximately 30% on the nonfasting days (7,30). Current guidelines for the management of overweight and obesity suggest that the minimal continuous daily energy deficit to promote weight loss should be ~500–750 kcal, corresponding to a total of 30% restriction from regular energy requirements (31). In the present study, subjects maintained unaltered their daily caloric intake during the first 2 months of intervention, whereas the drop in energy intake (approximately 180 kcal) was observed upon completion of 12 months of diet regimen. Unfortunately, we were unable to detect at which point, from months 2 to 12, subjects started to reduce their calories. However, weight loss is not the same over time during a dietetic regimen, and normally, the energy deficit needs to increase along with diet’s prolongation (32,33). Based on these considerations and because of the nature of research in free-living subjects, it is plausible that the caloric reduction observed in the TRE group may have contributed to the reductions in body mass and additional health benefits and implemented the effect of the fasting program.

The progressive decrement of RR at 2 and 12 months of the TRE diet suggests a shift toward greater reliance on fatty acids for fuel, whereas no change was observed during a normal diet protocol. Lower fasting RR (<0.75) is associated with a reduced risk of future weight gain (34,35). In the present study, although RR did not directly reach the recommended lower prognostic value of 0.75, our results suggest that a long-term TRE regimen may have a positive effect of future weight control.

Resistance training is a type of exercise program aimed at improving muscle strength and size, so it is not surprising that after an ND regimen, subjects increased muscle mass, thigh and arm CSA, and strength. Conversely, the combination of TRE with a resistance training program can maintain muscle mass in the short-term, although in the present long-term study, we observed a significant reduction in FFM, along with a significant reduction in limb CSA. Interestingly, TRE did not compromise muscle strength, which equally increased in both groups. These findings support a data published in a recent meta-analysis, which observed that the combination of RET with IF protocols promotes body mass and FM loss, but also underlie how these modifications are more evident after longer interventions (36).

The effect of TRE on muscle mass can be explained in part by the reduction in concentrations of anabolic hormones such testosterone and IGF-1. We previously reported a reduction in these two hormones in our short-term study (16); however, during the first 2 months of treatment, subjects in the TRE group did not restrict their energy intake, as during the 12 months. It has been previously described that CR can result in a decreased testosterone concentration (37), probably via a leptin influence on the hypothalamus–hypophysial–gonadal axis (38). Moreover, it is plausible that the effect of TRE on leptin and adiponectin and their influence on the AMPK/ACC pathway may also alter the GH/IGF-1 axis resulting in a decrease in IGF-1 concentration (39). With our results, we confirmed that a long-term TRE protocol could impair the ability of maintaining muscle mass, possibly because of a reduction in caloric intake and a direct effect of fasting on the production of anabolic hormones.

Adipose tissue is an important regulator of energy homeostasis, serving both as the site of storage of TG and an endocrine organ. The adipolysis observed in the TRE group may have been stimulated by adiponectin. Adiponectin is a cytokine involved in mitochondrial biogenesis through the activation of PGC-1α–AMPK kinase axis (40). Higher levels of this protein are associated with increased resting energy metabolism (40,41), whereas it inversely correlates with obesity (42). We have previously reported an increase in adiponectin after 8 wk of TRE (16); in the present study, we confirmed that this adaptation persists during long-term TRE. Moreover, the reduced level of this cytokine reached after 2 months correlated with body mass (*r* = −0.72, *P* = 0.0004) and FM (*r* = −0.52, *P* = 0.03) changes at 12 months, suggesting an important contributing role in weight control. Interestingly, leptin, the other hormone linked to body homeostasis and weight control, decreased with TRE. Leptin is synthetized in the adipose tissue and, once released, acts on the hypothalamus to reduce the stimulus of hunger and subsequent energy consumption. As previously observed (16), leptin decreased with TRE in the present study, but this difference did not persist when concentrations were normalized to FM. Leptin correlated with FM, and thus, this decrease reflected the loss of adipose tissue in TRE and cannot be attributed to the metabolic and endocrine adaptation to the diet protocol.

Adipose tissue can act as an endocrine organ and, as such, contributes to systemic regulation of glucose and lipid metabolism (43). The long-term effect of IF on glucose tolerance is still debated. Cerqueira and da Cunha (41) showed that long-term IF increases glycemia after feeding, resulting in an impaired glucose tolerance in rats. In the present study, TRE promoted a decrease in both blood glucose and insulin concentrations, resulting in a significant reduction in HOMA-IR index, a homeostatic model to assess insulin sensitivity. It has already been observed that IF (mostly a daily IF approach) consistently improved insulin sensitivity after several weeks of treatment in both obese and prediabetic subjects (7,44,45). From these studies, it seems that the improvements in insulin sensitivity were primarily associated with an important reduction in body mass; however, no correlation was found, either in the cited studies or in the present research, between visceral FM and insulin sensitivity changes. We instead observed a negative correlation between insulin sensitivity and adiponectin concentrations; this hormone is indeed known to have a pivotal role in combatting the development of insulin resistance and metabolic diseases by increasing tissue fat oxidation (46). Moreover, we found a significant decrease in the plasma LAR with TRE. The LAR has recently been proposed as a reliable marker to predict insulin resistance and vascular risk in healthy adults (47,48): when an adipocyte became hypertrophic, it tends to increase the secretion of leptin relative to adiponectin, increasing the ratio between these two adipokines. The reduction in LAR was positively correlated with the HOMA-IR adaptation at 2 and 12 months of diet protocol and was also associated with TG concentration changes after 12 months of intervention. In our previous study, we did not detect any significant differences for lipids profile, with the exception of TG. In a 12-month long-term study, Trepanowski and colleagues (49) compared the effect of an alternate day fasting protocol with daily CR diet on cardiometabolic risk factors in obese adults. In both protocols, the caloric intake was reduced by 25%; interestingly, in the mentioned study, the authors observed no differences between interventions in lipid profile, TGs, or fasting glucose and insulin. Taken together, these data suggest that a TRE protocol, especially if sustained for a longer period (>8 wk), may be a valid tool to reduce the risk of cardiometabolic disease and that this effect is not correlated with CR.

Likewise, it is possible that the improvement of insulin sensitivity could be related to the anti-inflammatory effect of adiponectin and other cytokines that influence numerous molecular pathways linked to glucose metabolism. Interestingly, using a subgroup of the original cohort from the 2016 study, we were not able to confirm the same significant effect of short-term TRE diet on TNF-α, IL-6, and IL-1β, probably because of a smaller sample size. In the original study, these markers were reduced, respectively, by 7.8% (*P* = 0.001), 16.7% (*P* = 0.0035), and 10.5% (*P* = 0.0042), whereas in the present subcohort, we observed a reduction in TNF-α of 8.3% (*P* = 0.0876), IL-6 of 24.4% (*P* = 0.0008), and IL-1β of 10.9% (*P* = 0.0643). However, all markers were significantly lower than baseline after 12 months of TRE, whereas no significant alteration was found with an ND regimen. Recently, McAllister et al. (50) showed reduced levels of cortisol, salivary C-reactive protein, IL-6, and IL-1β after an 8-wk TRE intervention (14:10 protocol) in professional firefighters. These findings suggest that the contradictory results of IF diets on inflammatory markers may be due to the different protocol and short duration of many studies and that longer treatment periods may be more beneficial for reducing inflammation in healthy adults.

One limitation of the present study is that the interventional caloric and macronutrient intake was based on interview, which is known to have some weakness. Moreover, diet was adjusted at 2 months, and adherence to diet protocol was assessed by phone regularly throughout the entire year of the intervention; however, this approach did not allow us to precisely prescribe the energy intake of participant. This led to a spontaneous reduction in total caloric and carbohydrates intake in TRE, which may have influenced the results of the study. It is worth noting that, although significant, a drop of 10% from energy requirement, corresponding to ~180 kcal, is not sufficient to promote such physiological adaptation in healthy adults. The European guidelines for obesity management in adults predict that a daily deficit of 600 kcal is needed to induce a weight loss of about 0.5 kg weekly (51), and the same range of energy restriction (500–700 kcal·d^−1^) is suggested to safely lose weight for athletes and active people. Moreover, CR could be a positive “collateral” effect for overweight subjects or those who need a severe control of dietary intake.

In summary, our results suggest that a 16:8 TRE protocol could be performed for longer period in trained individual with beneficial effects on health biomarkers and inflammatory indices. However, long-term TRE leads to a spontaneous CR, which can negatively affect muscle mass.
